# High-Normal Preconception TSH Levels Have No Adverse Effects on Reproductive Outcomes in Infertile Women Undergoing the First Single Fresh D5 Blastocyst Transfer

**DOI:** 10.1155/2020/1056484

**Published:** 2020-08-18

**Authors:** Yuchao Zhang, Wenbin Wu, Yanli Liu, Yichun Guan, Xingling Wang, Liting Jia

**Affiliations:** ^1^Department of Reproductive Medicine, The Third Affiliated Hospital of Zhengzhou University, Zhengzhou, Henan, China; ^2^Neonatal Screening Center, The Third Affiliated Hospital of Zhengzhou University, Zhengzhou, Henan, China

## Abstract

**Purpose:**

To investigate the association between high-normal preconception TSH levels and reproductive outcomes in infertile women undergoing the first single fresh D5 blastocyst transfer.

**Methods:**

This was a retrospective study. Euthyroid patients undergoing the first single fresh D5 blastocyst transfer from January 2018 to May 2019 were initially included. The patients were divided into a low TSH (0.27–2.5 mIU/L) group and a high-normal TSH (2.5–4.2 mIU/L) group. The reproductive outcomes were compared between the groups.

**Results:**

A total of 824 women were ultimately included, 460 of whom had serum TSH levels less than 2.5 mIU/L and 364 of whom had serum TSH levels between 2.5 and 4.2 mIU/L. The patients were highly homogeneous in terms of general characteristics. High-normal TSH levels had no adverse impact on the clinical pregnancy rate, miscarriage rate, or live birth rate (respectively, aOR = 0.84, 0.65, 0.61, and *P*=0.234, 0.145, 0.083). No significant differences were observed in terms of gestational age, single live birth rates, birth weight, or birth length.

**Conclusion:**

High-normal TSH levels did not significantly influence reproductive outcomes in infertile women undergoing the first single fresh D5 blastocyst transfer. Further studies are needed to test whether the results might be applicable to a wider population.

## 1. Introduction

Subclinical hypothyroidism (SCH) is defined as a thyrotropin (TSH) level higher than the upper limit of the normal range with normal free thyroxine (FT4) levels [1]. There is still debate regarding the definition of SCH (the threshold value might range from 2.5 to 5.5 mIU/L according to different studies) and the decision of when to treat, particularly for women attempting pregnancy [2]. When SCH was defined as a TSH level >4 mIU/L, there was fair evidence showing that it was associated with miscarriage and adverse obstetric outcomes. Experts suggested that TSH concentrations <2.5 mIU/L should be maintained during the ART procedure in infertile patients, especially those with overt hypothyroidism. However, there is insufficient evidence indicating an association between mildly elevated preconception TSH (2.5–4.0 mIU/L) and the abovementioned reproductive outcomes [3]. In recent years, studies investigating the impact of mildly elevated TSH levels on infertile women have emerged [4–14]. However, when infertile women underwent in vitro fertilization or intracytoplasmic sperm injection (IVF/ICSI) treatment, the conclusion of the impact of mildly elevated TSH levels on miscarriage and obstetric outcomes in women of reproductive age was still inconsistent in different studies, in which various confounders (such as the transfer of cleavage embryo or blastocysts, the number and quality of the transferred embryo, and the age of included women) influenced the reproductive outcomes. For that reason, the aim of this study was to evaluate the impact of preconception TSH levels between 2.5 and 4.2 mIU/L (which was used as the upper limit of the reference range in our laboratory) on reproductive outcomes in women undergoing their first single fresh blastocyst transfer.

## 2. Population and Methods

### 2.1. Study Population

A total of 1056 infertile women receiving fresh blastocyst transfer in the Department of Reproductive Medicine, the Third Affiliated Hospital of Zhengzhou University, from January 2018 to May 2019, were initially included. Generally, the history of infertility in infertile couples is complex, most of which are either male factors or pelvic and fallopian tube factors or both. The women underwent standard controlled ovarian hyperstimulation (COH) with a downregulation protocol using a GnRh agonist or an antagonist protocol according to the patients' conditions. Oocytes were retrieved transvaginally 34–36 h after HCG administration and were inseminated using either the IVF or the ICSI method. Rescued ICSI was performed when necessary to avoid failure of insemination. Variable blastocysts were developed starting on the 5^th^ day after insemination and were scored according to the Gardner system. The blastocyst was transferred to the uterus under the guidance of ultrasound. Patients with the following criteria were excluded: D6 blastocyst was transferred (*n* = 3); more than one blastocyst was transferred (*n* = 1); previous embryo transfers (*n* = 79); the presence of overt or subclinical thyroid dysfunction (*n* = 124); and lack of essential data (*n* = 24). After exclusions, 824 women were transferred with a single D5 blastocyst at the first IVF/ICSI cycle and were divided into two groups based on preconception TSH levels (low TSH group, 0.27–2.5 mIU/L, *n* = 460; high-normal TSH group, 2.5–4.2 mIU/L, *n* = 364). The study was carried out in accordance with the Code of Ethics of the Declaration of Helsinki.

### 2.2. Laboratory Tests

Before sample collection, the patients were required to stay calm for at least 30 minutes. All fasting blood samples were collected from patients on the 2^nd^–4^th^ morning of menstruation. Samples were centrifuged at 3000 rpm for 10 minutes after half an hour. Then, the serum on the upper layer was used for analysis. TSH, FT3, and FT4 were measured as part of the infertility workup before fertility treatment for first visitors. The measurements were conducted by electrochemical luminescence (ECLIA) on a Cobas 8000 (Roche Diagnostics, Germany). Generally, thyroid function tests were performed no longer than six months before blastocyst transfer. The provided TSH reference range is 0.27–4.2 mIU/L, the FT3 reference range is 3.1–6.8 pmol/L, and the FT4 reference range is 12.0–22.0 pmol/L. Daily internal quality control and yearly external quality control were carried out by request.

### 2.3. Definition of Clinical Outcomes

Pregnancy was defined as positive serum *β*-hCG (>10 IU/L). Clinical pregnancy was defined as the presence of the gestational sac and fetal heart activity following positive serum *β*-hCG. Early embryo loss was defined as a lack of visible gestational sac or fetal heart activity following positive serum *β*-hCG. Ectopic pregnancy was defined as a pregnancy that did not occur inside the uterine cavity. Miscarriage, which included early abortion, intermediate abortion, and late abortion, was defined as a pregnancy that did not result in delivery. Preterm birth was defined as a gestational age less than 259 days (37 weeks). Live birth was defined as the delivery of live babies.

### 2.4. Statistical Analyses

Our primary outcomes were clinical outcomes, such as clinical pregnancy rate, early pregnancy loss rate, miscarriage rate, and live birth rate. The secondary outcomes were obstetric outcomes, such as the single live birth rate, birth weight, birth length, and gestational age. Continuous data are expressed as the mean (SD), and statistical comparisons were performed by either Student's *t*-test or the Mann–Whitney *U* test where appropriate. Categorical variables were expressed as numbers or percentages, and the chi-square test or Fisher's exact test was used for comparisons of categorical variables where appropriate. Binary logistic regression was then used to evaluate the impact of preconception high-normal TSH levels on the clinical and obstetric outcomes. Data were analyzed using SPSS 22.0 statistical software. All *P* values less than 0.05 were considered statistically significant.

## 3. Results

### 3.1. General Characteristics of the Included Women

**Figure 1:**
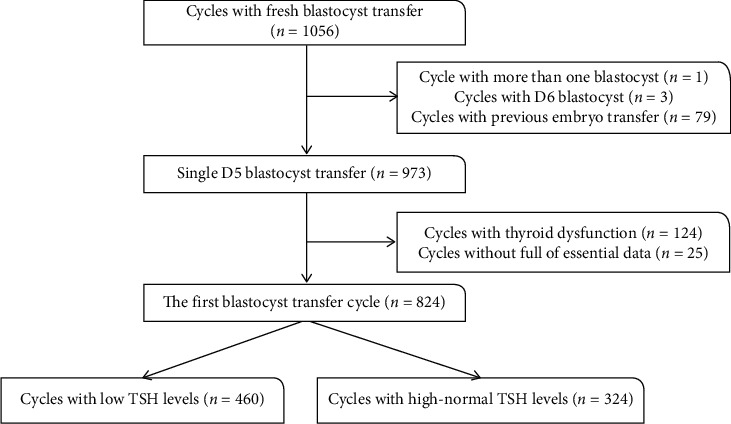
Flow chart of the inclusion of women undergoing the first single blastocyst transfer treatment.

The flowchart of the included women is shown in Figure 1. The results predicted that a cohort of highly homogeneous women were included. Specifically, there were no differences between the TSH groups in terms of age, BMI, AMH, FT4, endometrial thickness, duration of infertility, and basal FSH (Table 1). Furthermore, there were no differences in the frequencies of the causes of infertility between the two groups (*P*=0.279). The frequencies of insemination methods and top quality of transferred blastocysts were similar in both groups (*P*=0.542). No differences were noted in terms of the type of infertility or scheme of ovarian stimulation (*P*=0.292 and 0.998, respectively). As hysterosalpingography (HSG) affects thyroid function, we further compared the proportion of patients who underwent HSG between the two groups and found a similar proportion (*P*=0.885).

In addition, we also compared the general characteristics of pregnant and nonpregnant women. As shown in Table 2, couples with a history of infertility caused by male factors were more likely to be pregnant (*P*=0.036). The mean TSH levels and age were also comparable between pregnant and nonpregnant women (*P*=0.260 and 0.514, respectively).

### 3.2. Clinical Outcomes of the Women Undergoing the First Single D5 Blastocyst Transfer

**Figure 2:**
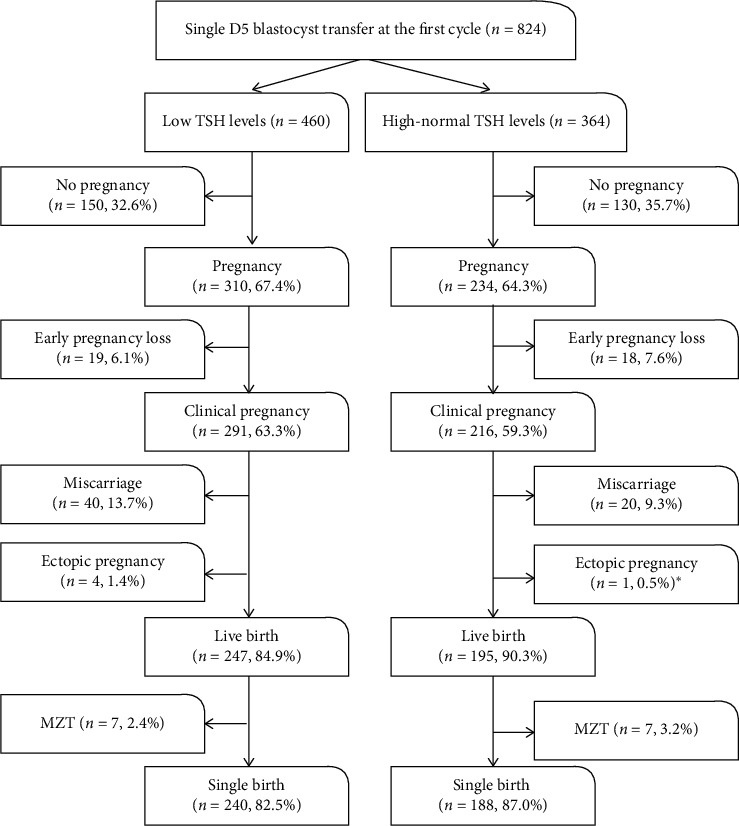
Clinical outcomes after the first single D5 blastocyst transfer. ^*∗*^Fisher's exact test was used.

As shown in Figure 2, comparisons of pregnancy rate, clinical pregnancy rate, live birth rate, and single live birth rate were performed between the low and high-normal TSH groups. There were no significant differences in the abovementioned reproductive outcomes between the two groups. In addition, there were no significant differences in the early pregnancy loss rate, miscarriage rate, or ectopic pregnancy rate between the two groups. Furthermore, binary logistic regression analysis between the groups indicated that AMH and quality of transferred blastocysts positively predicted the clinical pregnancy rate, with adjusted ORs of 1.01 (*P*=0.049) and 1.61 (*P*=0.006), respectively, while younger age had significantly positive effects on the miscarriage rate and live birth rate, with adjusted ORs of 0.91 and 1.10 (*P*=0.015 and 0.005), respectively. High-normal TSH levels had no adverse impact on the abovementioned outcomes (aOR = 0.84, 0.65, 0.61, and *P*=0.234, 0.145, 0.083 for clinical pregnancy rate, miscarriage rate, and live birth rate, respectively) (Table 3).

### 3.3. Obstetric Outcomes of the Women Undergoing the First Single D5 Blastocyst Transfer

We further compared obstetric outcomes between the low and high-normal TSH groups. Of the 824 included women, 247 in the low TSH group had live births, while 195 in the high-normal TSH group had live births (Table 4). No significant difference was observed in terms of gestational age (*P*=0.622). The preterm birth rates were similar in both groups (8.1% vs. 8.2%). The single live birth rates were similar between the low and high-normal TSH groups (preterm birth, *P*=0.712; full-term birth, *P*=0.409). In addition, the birth weight and birth length of single live births were similar between the low and high-normal TSH groups (*P*=0.311 and 0.921, respectively), as were the birth weight and length of twin live births (*P*=0.159 and 0.602, respectively).

## 4. Discussion

In this retrospective study, we investigated the association between preconception TSH and the reproductive outcomes of 824 infertile women undergoing the first single D5 blastocyst transfer at a university-affiliated hospital and found that a high-normal preconception TSH level (between 2.5 and 4.2mIU/L) was not associated with lower odds of clinical pregnancy and live birth or higher odds of miscarriage. Our results were consistent with those of some previous studies, as the debate regarding the impact of high-normal TSH on ART clinical outcomes still exists.

Based on a population of more than eighteen thousand naturally pregnant women in China, Chen et al. clarified that preconceptionally high TSH levels were associated with a small but significantly increased risk of overall adverse events, even within the normal unpregnant range, and concluded that TSH <2.5 mIU/L was more suitable for the assessment of women planning for pregnancy [15]. However, the impact of preconception TSH in women undergoing fertility treatment is conflicting.

Our previous results were in agreement with those of other studies, which demonstrated that mildly elevated preconception TSH levels before treatment had no adverse impact on the clinical outcomes in infertile women undergoing intrauterine insemination (IUI) treatment [6, 9, 12]. In this study, we further included a cohort of highly homogeneous infertile women who were transferred with a single fresh D5 blastocyst and found no significant differences between the low and high-normal TSH groups in terms of clinical pregnancy rate, early pregnancy rate, miscarriage rate, and live birth rate. Similarly, in a large retrospective cohort study of first-cycle IVF patients, Reh et al. used a TSH cutoff of 2.5 mIU/L or 4.5 mIU/L, and no differences in the rates of clinical pregnancy, delivery, or miscarriage were observed after adjustments were made for age [16]. Marziyeh et al. reported a lack of association between TSH levels in the range of 0.5–4.5 mIU/L and clinical pregnancy rate and stated that lowering the upper limit of normal TSH should still be considered a scientific debate. Chai et al. also reported that the clinical pregnancy rate and miscarriage rate were not impaired when the TSH level was high-normal [8]. In agreement with our results, So et al. and Unuane et al. both claimed that cumulative pregnancy rates were similar between women with low TSH levels and those with high-normal TSH levels [4, 7].

According to the molecular basis of TSH and thyroid hormone action during implantation and early development, the thyroid hormone might be involved in endometrium preparation for pregnancy and initial trophoblast development, and thyroid hormones are essential players in the mechanisms regulating implantation and early fetal development [17]. Indeed, Unuane et al. and we both found no difference in FT4 levels between low and high-normal TSH groups, which may partially explain the similar results between the two groups [7, 12].

In contrast, Grove Laugesen et al. claimed a detrimental effect of high-normal preconception TSH levels on the clinical outcomes of infertile women undergoing their first IVF cycle [10]. In our study, a high-normal TSH level had no adverse effect on clinical pregnancy rates, miscarriage rates, and live birth rates, regardless of adjustment for confounders such as age, AMH, quality of blastocyst, and causes of infertility. Discrepancies might be explained by the differences in the included population and the transferred embryos. Specifically, the proportion of women in the high-normal TSH group was much higher in our study (44.2% vs. 18.3%). Notably, the prevalence of SCH (TSH cutoff: 4.2 mIU/L) in the general population in China was 16.7%, which was close to the proportion of the population with high-normal TSH levels [18]. In addition, the women in our study were transferred with a single fresh blastocyst, while details about the transferred embryos in the study by Grove Laugesen et al. were not reported.

Baker et al. further demonstrated that a preconception TSH level >2.5 mIU/L was associated with a lower gestational age at delivery and lower birth weight in women undergoing IVF treatment [19]. It should be noted that the study performed by Baker et al. included a population with a TSH level >4.0 mIU/L, which is currently used as the cutoff to define SCH. Our study included women with TSH values lower than the upper limit of the reference range. The results showed no differences in terms of gestational age, birth weight, and birth length between the low and high-normal TSH groups, and the differences existed when we controlled for twin live births. Furthermore, we compared the incidence of preterm birth between the low and high-normal TSH groups and found similar results, which supported the observations found in women undergoing their first IUI treatment.

There were also some limitations in this study. First, this was a retrospective study with data collected from a single center, and selection bias was inevitable. Second, we did not report the status of thyroid antibodies in infertile women, which might have a role in infertility and miscarriage. However, on the one hand, the existing data are controversial regarding whether thyroid antibodies are associated with infertility or adverse reproductive outcomes [6–8, 13, 20]. On the other hand, according to the two RCTs of high quality, euthyroid women with positive TPO did not benefit from treatment with LT4 [21, 22]. Third, we included a cohort of infertile women with specified conditions, such as younger age with good ovarian reserve, which we believed to be one of the crucial factors explaining the discrepancies in the results. Whether the results might be applied to women with D3 embryo transfer, women with repeated embryo transfer or healthy women remain to be tested. Finally, we did not monitor the TSH level once it was within the normal range before treatment. It was confirmed in other studies that high-normal TSH was more prone to increase to a level higher than the upper limit of normal range during ovarian stimulation and decrease within the normal range after embryo transfer [23–27]. In addition, TSH >2.5 mIU/L in early pregnancy contributes to miscarriage. Whether temporarily increased TSH levels might affect clinical outcomes remains unknown.

The strength of our study was the highly homogeneous population-based cohort of euthyroid women, as the number, stage, or quality of transferred embryos, as well as the age and ovarian reserve, might potentially affect the results of embryo transfer. It was reported that HSG affected thyroid function [28]. Here, in our department, our patients underwent embryo transfer more than a month after HSG with the water-contrast medium. We believe that the impact of HSG on the thyroid should not be considered. We also compared the differences in obstetric outcomes, such as gestational age, birth weight, and birth length, between the low- and high-normal TSH groups and found that regardless of TSH levels within the normal range, AMH or blastocyst quality was independently associated with clinical pregnancy and that in those who were pregnant, advanced age was independently associated with lower odds of live birth and higher odds of miscarriage.

In conclusion, we included infertile women as homogeneous as possible to minimize the bias that may lower the power of the impact of TSH on reproductive outcomes. We found that a high-normal TSH level did not significantly influence the reproductive outcomes of infertile women undergoing the first single fresh D5 blastocyst transfer. Further studies are needed to test whether the results might be applicable to a wider population.

## Figures and Tables

**Table 1:** The general characteristics of the included women based on TSH levels.

	TSH (0.27–2.5)	TSH (2.5–4.2)	*P*
*N*	460	364	
Age (years)	29.92 (4.02)	29.98 (3.82)	0.839
BMI (kg/m^2^)	23.40 (3.09)	23.78 (3.26)	0.093
AMH (pmol/L)	34.68 (20.04)	37.40 (24.68)	0.082
FT4 (pmol/L)	16.62 (2.01)	16.47 (2.10)	0.248
Basal FSH (IU/L)	6.70 (1.77)	6.88 (1.68)	0.142
Endometrial thickness (mm)	11.66 (2.24)	11.80 (2.28)	0.384
Duration of infertility (year)	3.11 (2.38)	3.37 (2.52)	0.132
Number of retrieved oocytes	17.34 (4.51)	17.73 (4.75)	0.229
*Insemination method of transferred blastocyst*			0.542
IVF	363 (78.9%)	294 (80.8%)	
ICSI	97 (21.1%)	70 (19.2%)	
*Quality of transferred blastocyst*			0.189
Top quality	359 (78.0%)	269 (73.9%)	
Nontop quality	101 (22.0%)	95 (26.1%)	
*Ovarian stimulation protocol*			0.998
GnRH agonist	449 (97.6%)	356 (97.8%)	
GnRH antagonist	11 (2.4%)	8 (2.2%)	
*Type of infertility*			0.292
Primary	218 (47.4%)	186 (51.1%)	
Secondary	242 (52.6%)	178 (48.9%)	
*Causes of infertility*			0.279
Male factors	102 (22.2%)	78 (21.4%)	
Female mixed factors	26 (5.7%)	17 (4.8%)	
Ovulation failure	34 (7.3%)	31 (8.5%)	
Pelvic and fallopian tube factors	168 (36.5%)	110 (30.2%)	
Factors on both sides	101 (22.0%)	98 (26.9%)	
Unexplained	29 (6.3%)	30 (8.2%)	
*Examination of uterine and fallopian tubes*			0.885
Hysterosalpingography	269 (58.5%)	208 (57.1%)	
Four-dimensional contrast enhanced ultrasound	11 (2.4%)	10 (2.7%)	
Hysteroscopy	97 (21.1%)	84 (23.1%)	
Others	83 (18.0%)	62 (17.0%)	

**Table 2:** General characteristics between pregnant and nonpregnant women.

	Nonpregnant	Pregnant	*P*
*N*	317	507	
Age (years)	30.06 (4.15)	29.88 (3.79)	0.514
BMI (kg/m2)	23.36 (3.07)	23.7 (3.23)	0.136
AMH (pmol/L)	34.13 (20.9)	36.97 (22.98)	0.074
TSH (mIU/L)	2.43 (0.88)	2.36 (0.83)	0.260
FT4 (pmol/L)	16.45 (2.05)	16.61 (2.04)	0.282
Basal FSH (IU/L)	6.79 (1.75)	6.78 (1.73)	0.943
Endometrial thickness (mm)	11.56 (2.21)	11.82 (2.28)	0.108
Duration of infertility (year)	3.36 (2.56)	3.15 (2.37)	0.231
Number of retrieved oocytes	17.85 (4.46)	17.31 (4.7)	0.099
*Insemination method of transferred blastocyst*			0.350
IVF	258 (81.4%)	399 (78.7%)	
ICSI	59 (18.6%)	108 (21.3%)	
*Quality of transferred blastocyst*			0.005
Top quality	225 (71%)	403 (79.5%)	
Nontop quality	92 (29%)	104 (20.5%)	
*Ovarian stimulation protocol*			0.078
GnRH agonist	306 (96.5%)	499 (98.4%)	
GnRH antagonist	11 (3.5%)	8 (1.6%)	
*Type of infertility*			0.219
Primary	164 (51.7%)	240 (47.3%)	
Secondary	153 (48.3%)	267 (52.7%)	
*Infertility treatments*			
None	209 (65.9%)	367 (72.4%)	0.268
IUI once	50 (15.8%)	65 (12.8%)	
IUI twice	35 (11%)	47 (9.3%)	
IUI more than 3 times	23 (7.3%)	28 (5.5%)	
*Causes of infertility*			
Male factors	62 (19.6%)	118 (23.3%)	0.036
Female mixed factors	14 (4.4%)	29 (5.7%)	
Ovulation failure	27 (8.5%)	38 (7.5%)	
Pelvic and fallopian tube factors	116 (36.6%)	162 (32%)	
Factors on both sides	66 (20.8%)	133 (26.2%)	
Unexplained	32 (10.1%)	27 (5.3%)	

**Table 3:** The impact of preconception high-normal TSH levels on clinical outcomes.

	Clinical pregnancy		Miscarriage		Live birth	
	aOR (95% CI)	*P*	aOR (95% CI)	*P*	aOR (95% CI)	*P*
TSH (low vs. high-normal level)	0.84 (0.63–1.12)^a^	0.234	0.65 (0.37–1.16)^a^	0.145	0.61 (0.34–1.05)^a^	0.083
Age (years)	0.99 (0.96–1.04)	0.941	0.92 (0.85–0.98)^b^	0.015	1.10 (1.03–1.19)^b^	0.005
AMH (pmol/L)	1.01 (1.00–1.09)^c^	0.049	1.01 (0.99–1.02)	0.448	0.99 (0.98–1.01)	0.441
Quality of blastocyst	1.61 (1.16–2.23)^d^	0.006	1.02 (0.52–2.02)	0.949	1.39 (0.75–2.59)	0.341
Causes of infertility	0.90 (0.76–1.07)	0.223	0.87 (0.63–1.22)	0.421	0.86 (0.62–1.19)	0.865

^a^High-normal TSH levels had no impact on clinical pregnancy, miscarriage, or live birth. ^b^Younger age predicted lower odds of miscarriage and higher odds of live birth. ^c^Higher serum AMH predicted very slightly higher odds of clinical pregnancy. ^d^Top quality blastocysts predicted higher odds of clinical pregnancy.

**Table 4:** The obstetric outcomes of the women undergoing the first single D5 blastocyst transfer.

		Low TSH group (*n* = 247)	High-normal TSH group (*n* = 195)	*P*
Gestational age (day)		272.6 (10.27)	273.0 (12.72)	0.622
Gestational age (days)	Single	273.4 (8.8)	274.3 (10.1)	0.301
Gestational age (days)	Twin	246.8 (18.7)	250.1 (15.6)	0.732
Preterm birth	Single	15 (75.0%)	13 (81.3%)	0.712
	Twin	5 (25.0%)	3 (18.7%)	
Full-term birth	Single	225 (99.1%)	175 (97.7%)	0.409
	Twin	2 (0.9%)	4 (2.3%)	
Birth weight	Single birth (g)	3391.3 (517.0)	3442.4 (522.5)	0.311
	Twin (g)	2129.3 (528.6)	2409.3 (368.5)	0.159
Birth length	Single birth (cm)	50.4 (1.59)	50.4 (1.9)	0.921
	Twin (cm)	46.9 (3.50)	46.1 (4.3)	0.602

## Data Availability

The original data are available to all readers upon request to the first author via mail (yuchao1988@yeah.net).
